# Immunomodulatory activity of *Tityus serrulatus* scorpion venom on human T lymphocytes

**DOI:** 10.1186/s40409-015-0046-3

**Published:** 2015-11-11

**Authors:** Andrea Casella-Martins, Lorena R Ayres, Sandra M Burin, Fabiana R Morais, Juliana C Pereira, Lucia H Faccioli, Suely V Sampaio, Eliane C Arantes, Fabiola A Castro, Luciana S Pereira-Crott

**Affiliations:** Department of Clinical Analyses, Toxicology and Food Sciences, School of Pharmaceutical Sciences of Ribeirão Preto, University of São Paulo (USP), Avenida do Café, s/n, Ribeirão Preto, SP CEP 14040-903 Brazil; Department of Pharmaceutical Sciences, Federal University of Espírito Santo, Vitória, ES Brazil; Department of Physics and Chemistry, School of Pharmaceutical Sciences of Ribeirão Preto, University of São Paulo (USP), Ribeirão Preto, SP Brazil

**Keywords:** Immunomodulation, Immunophenotyping, T lymphocyte, Cell proliferation, Cytokine, *Tityus serrulatus* venom

## Abstract

**Background:**

*Tityus serrulatus* scorpion venom (TsV) contains toxins that act on K^+^ and Na^+^ channels and account for the venom’s toxic effects. TsV can activate murine peritoneal macrophages, but its effects on human lymphocytes have been poorly investigated. Considering that lymphocytes may play an important role in envenomation, we assessed whether TsV affects the expression of phenotypic (CD3, CD4, and CD8) and activation (CD69, CD25, and HLA-DR) markers, cell proliferation, and cytokine production in peripheral blood mononuclear cells.

**Methods:**

Cytotoxicity of TsV was evaluated via the MTT assay. Cell proliferation, expression of phenotypic and activation markers, and release of cytokines were assessed using flow cytometry, after treatment with non-cytotoxic concentrations of TsV. The combined use of carboxyfluorescein diacetate succinimidyl ester and monoclonal antibodies against phenotypic and activation markers enabled us to simultaneously assess cell proliferation extent and cell activation status, and to discriminate among cell subpopulations.

**Results:**

TsV at concentrations of 25 to 100 μg/mL were not cytotoxic towards peripheral blood mononuclear cells. TsV did not induce significant changes in lymphocyte subpopulations or in the expression of activation markers on CD4^+^ and CD8^+^ T cells. TsV inhibited the phytohemagglutinin-stimulated lymphocyte proliferation, particularly in the CD8^+^ CD25^+^ T lymphocyte subset. TsV alone, at 50 and 100 μg/mL, did not induce peripheral blood mononuclear cell proliferation, but elicited the production and release of IL-6, a proinflammatory cytokine that plays an important role in innate and adaptive immune responses.

**Conclusions:**

TsV is a potential source of molecules with immunomodulatory action on human T lymphocytes.

**Electronic supplementary material:**

The online version of this article (doi:10.1186/s40409-015-0046-3) contains supplementary material, which is available to authorized users.

## Background

Scorpions from the family *Buthidae* are responsible for the majority of worldwide envenomations, especially in South Asia, the Middle East, Central and South America, and North Africa [1, 2]. *Tityus* is one genus of this family, of which *Tityus serrulatus* is the most dangerous species – it causes serious envenomation that may provoke fatalities, the majority of the victims are children [3–7].

Scorpion venom is composed of numerous toxins, mostly of peptides and neurotoxins, which act by deregulating cell membrane ion channels. Such toxins may cause pain, deregulation of cardiovascular and autonomic nervous systems, vomiting, abdominal pain, stimulation of peripheral nervous system, neuromuscular excitation, among other deleterious effects in humans [8].

Massive activation of the immune system seems also to participate in the pathogenesis of *T. serrulatus* envenomation. The plasma levels of NO and cytokines – such as interleukin-1 (IL-1), IL-6, IL-8 and interferon-γ (IFN-γ) produced by immune cells – are increased in patients presenting moderate or severe *T. serrulatus* envenomation, and correlate with the severity of envenomation [9]. The participation of proinflammatory cytokines in the moderate and severe envenomation pathophysiology indicates that these patients may benefit from treatment with glucocorticoids.

Recent studies reported that the venom of *T. serrulatus* and its toxins activate murine macrophages, which are critical cells for the immune response because they participate in humoral and cellular responses [10–15]. However, the effect of *Tityus serrulatus* scorpion venom (TsV) on human lymphocytes remains to be investigated. Considering that TsV contains several toxins that act on membrane K^+^ channels, and that these channels are involved in the regulation of cellular functions, in the present study we examined whether TsV interferes in peripheral blood mononuclear cell (PBMC) cytokine production, as well as in T lymphocyte phenotype, proliferation, and expression of activation markers.

## Methods

### Source and preparation of venom

TsV was obtained from the serpentarium of the Ribeirão Preto Medical School, University of São Paulo, Brazil, by using the electrical stimulation method with the minimal voltage required (− 12 V) [16].

Lyophilized TsV (4 mg) was diluted with 10 mL of sterile distilled water and fractionated by centrifugation (10,015 × g, 4 °C, 10 min), yielding an insoluble and non-toxic fraction composed of mucoproteins and membrane debris. The soluble part was rich in basic neurotoxic proteins, but also contained various enzymes and organic (lipids, carbohydrates, nucleosides, free amino acids) and inorganic (ions) compounds. Protein concentrations in crude soluble venom were determined by absorbance readings at 280 nm using a NanoDrop 2000 spectrophotometer (Thermo Scientific, Wilmington, DE, USA) and the extinction coefficient of the soluble venom [17]:

$$ {\upvarepsilon}_{280\ \mathrm{nm}}^{1\ \mathrm{mg}/\mathrm{mL}} = 1.65 $$

### Peripheral blood mononuclear cell (PBMC) isolation and culture

PBMC were isolated from venous blood of 20 healthy adult volunteers (7 males and 13 females, 20–40 years old) via the Ficoll-Hypaque (Hystopaque®-1077; Sigma-Aldrich, USA) discontinuous density centrifugation method. PBMC were washed twice, suspended in RPMI 1640 medium (GIBCO®, USA) to a concentration of 5 × 10^6^ cells/mL. Cells were cultured in RPMI with phytohemagglutinin (PHA; 2 μg/mL; Sigma-Aldrich, USA) and/or TsV (25, 50, and 100 μg/mL) at 37 °C and under 5 % CO_2_, during the proliferation assay (96 h) and before the immunophenotyping assay (24 h). The Human Research Ethics Committee at FCFRP-USP approved the research protocol, registered under number CEP/FCFRP 166.

### Cytotoxicity evaluation

The cytotoxic activity of TsV was evaluated using the MTT method described by Mosmann [18]. PBMC (5 × 10^5^ cells/well) were cultured in 96-well plates in RPMI 1640 complete culture medium containing 10 % fetal calf serum (FCS) in the presence of TsV (12.5; 25.0; 50.0; 62.5; 100.0; 125.0; 250.0; 500.0; 1000.0; 2000.0 μg/mL) or cyclophosphamide (Cyclo; 25 mg/mL; positive control; Sigma-Aldrich, USA) previously diluted in culture medium, for 24 h at 37 °C, and under 5 % CO_2_. Next, 20 μL of MTT (Sigma-Aldrich, USA) solution at 5 mg/mL was added to each well, and the plates were incubated for 4 h, at 37 °C, and under 5 % CO_2_. After incubation, the precipitated formazan crystals were dissolved with 200 μL of 20 % SDS (sodium dodecylsulfate), and absorbance was recorded at 570 nm. Absorbance values recorded for untreated cells (negative control) represent 100 % of PBMC viability, and were used as reference to calculate the percentage of cell viability in the presence of each sample concentration.

### Immunophenotyping of lymphocyte subsets and activation markers

Flow cytometry analysis was performed to identify and quantify lymphocyte subpopulations (CD3-FITC, CD4-PE, and CD8-PE monoclonal antibodies), and to measure the levels of cellular activation markers expression (CD69, CD25, and HLA-DR monoclonal antibodies conjugated with APC) (Becton-Dickinson, USA).

PBMC (2 × 10^5^ cells per well) were cultured with TsV (25; 50; 100 μg/mL) for 24 h at 37 °C, and under 5 % CO_2_. Cells were collected, suspended to a concentration of 1 × 10^6^ cells in 100 μL of FACS buffer (phosphate buffered saline – PBS, with 10 % FCS and 1 % sodium azide), and incubated with 1 μL of monoclonal antibodies at the following concentrations (mg/mL): 0.0031(CD4); 0.0125 (CD8); 0.012 (CD25); 0.2 (CD69); 0.05 (HLA-DR); and 0.1 (CD3), for 30 min at 4 °C, in the dark. Then, the cells were washed with PBS and suspended in 100 μL of PBS-FCS.

A total of 30,000 events per tube was acquired on the flow cytometer (FACSCanto, Becton-Dickinson, USA), the lymphocyte gate was selected according to its forward and side scatter distribution, and further analyzed with the aid of the software FACSDiva (Becton-Dickinson, USA). The results were expressed as percentages of stained cells. Additional file 1 depicts representative forward/side scatter dot-plots of human PBMC treated with TsV.

### Cell proliferation assay

The use of carboxyfluorescein diacetate succinimidyl ester (CFSE) in combination with monoclonal antibodies against phenotypic and activation markers enabled the concomitant determination of cell proliferation and activation status and discrimination of cell subpopulations.

PBMC were centrifuged, suspended to the concentration of 5 × 10^6^ cells/mL in PBS containing 0.1 % human albumin, and labeled with CFSE (2.5 μM; Molecular Probes, USA) for ten minutes at 37 °C. The labeling process was stopped by addition of five volumes of ice-cold RPMI 1640 containing 10 % FCS (RPMI-FCS) followed by incubation for five minutes in an ice bath, in the dark. The cells were washed three times with 20 mL of RPMI-FCS, and further suspended in the same medium.

PBMC were plated in 96-well plates (5 × 10^5^ cells/well) and cultured with PHA (2 μg/mL) and/or TsV(25, 50, or 100 μg/mL) for 96 h at 37 °C, and under 5 % CO_2_. The cells (1 × 10^6^) were transferred to flow cytometry tubes and labeled with 2 μL of the following monoclonal antibodies: anti-CD3/PerCP (Peridinin-chlorophyll-protein complex); anti-CD8, or anti-CD4 PE; anti-CD25, anti-HLA-DR, or anti-CD69 APC (Becton-Dickinson, USA). A total of 30,000 events per tube was acquired on the flow cytometer (FACSCanto, Becton-Dickinson, USA) and analyzed with the aid of the software FACSDiva (Becton-Dickinson, USA). The lymphocyte gate was set on light-scatter properties (forward scatter vs. side scatter). The results were expressed as percentages of stained cells.

### Cytokine quantification

The levels of tumor necrosis factor-α (TNF-α), IL-2, IL-4, IL-6, IL-10, IL-17, and IFN-γ in culture supernatants of PBMC (1.0 × 10^5^ cells) treated with PHA (2 μg/mL) and/or TsV (25, 50, and 100 μg/mL) for 96 h were quantified by flow cytometry, using the assay kit Cytometric Bead Array (CBA) Human Th1/Th2/Th17 Cytokine (BD Biosciences, USA) according to the manufacturer’s instructions. The samples were analyzed on a FACSCanto cytometer with the aid of the CBA analysis software FCAP Array (version 1.01; Becton-Dickinson, USA). The cytokine concentration was expressed as pg/mL.

### Statistical analysis

The results were expressed as mean ± standard error of the mean (SEM). Statistical analysis was performed by one-way analysis of variance (ANOVA) followed by the Tukey’s test, using the software Graph Pad Prism 5. Significance was defined as *p* < 0.05.

## Results

### TsV at lower concentrations is not cytotoxic towards PBMC

**Fig. 1:**
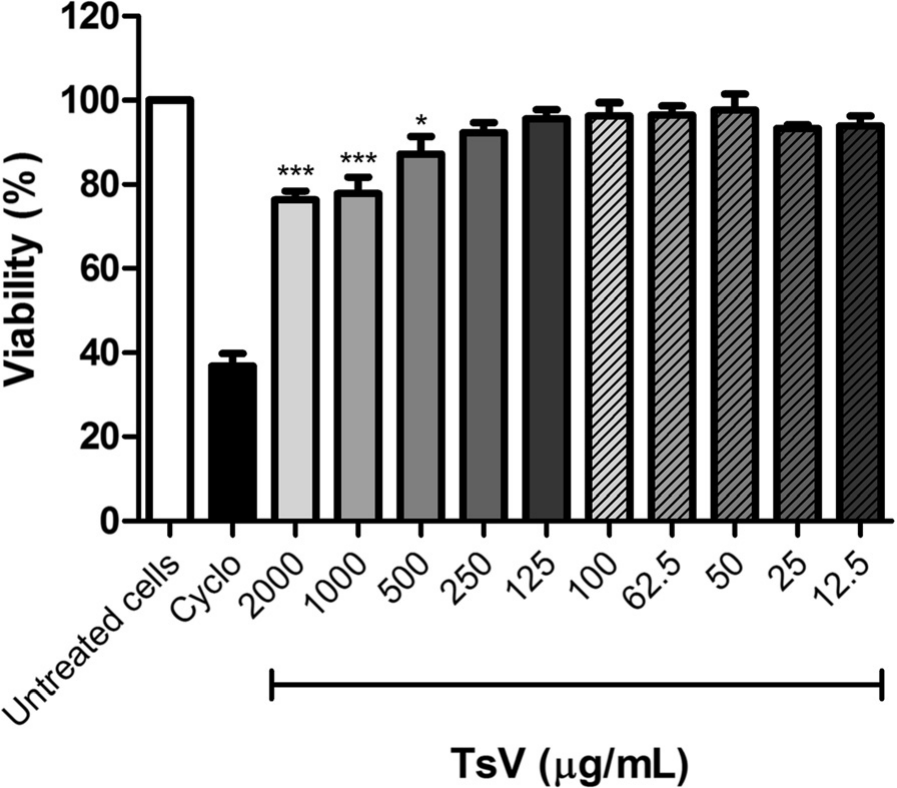
Effect of *Tityus serrulatus* scorpion venom (TsV) on peripheral blood mononuclear cell (PBMC) viability. PBMC (5 × 10^5^ cells/well) were treated with different concentrations of TsV for 24 h, and cell viability was assessed by the MTT method. Cyclophosphamide (Cyclo; 25 mg/mL) was used as positive control. Results are expressed as mean (± SEM) percentage of cell viability calculated in relation to untreated cells (*n* = 6 experiments). *p < 0.05 and ***p < 0.001 vs. untreated cells

We used the MTT assay to examine whether TsV is cytotoxic towards PBMC. Compared with untreated cells, TsV at concentrations of 500, 1000, and 2000 μg/mL reduced cell viability by 12.7, 22.2, and 23.7 %, respectively. TsV was not cytotoxic towards PBMC at concentrations lower than 250 μg/mL (Fig. 1). Based on these results, we selected the TsV concentrations of 25, 50, and 100 μg/mL for further experiments.

### TsV does not induce changes in T lymphocyte subpopulations and in the expression of PBMC activation markers

**Fig. 2:**
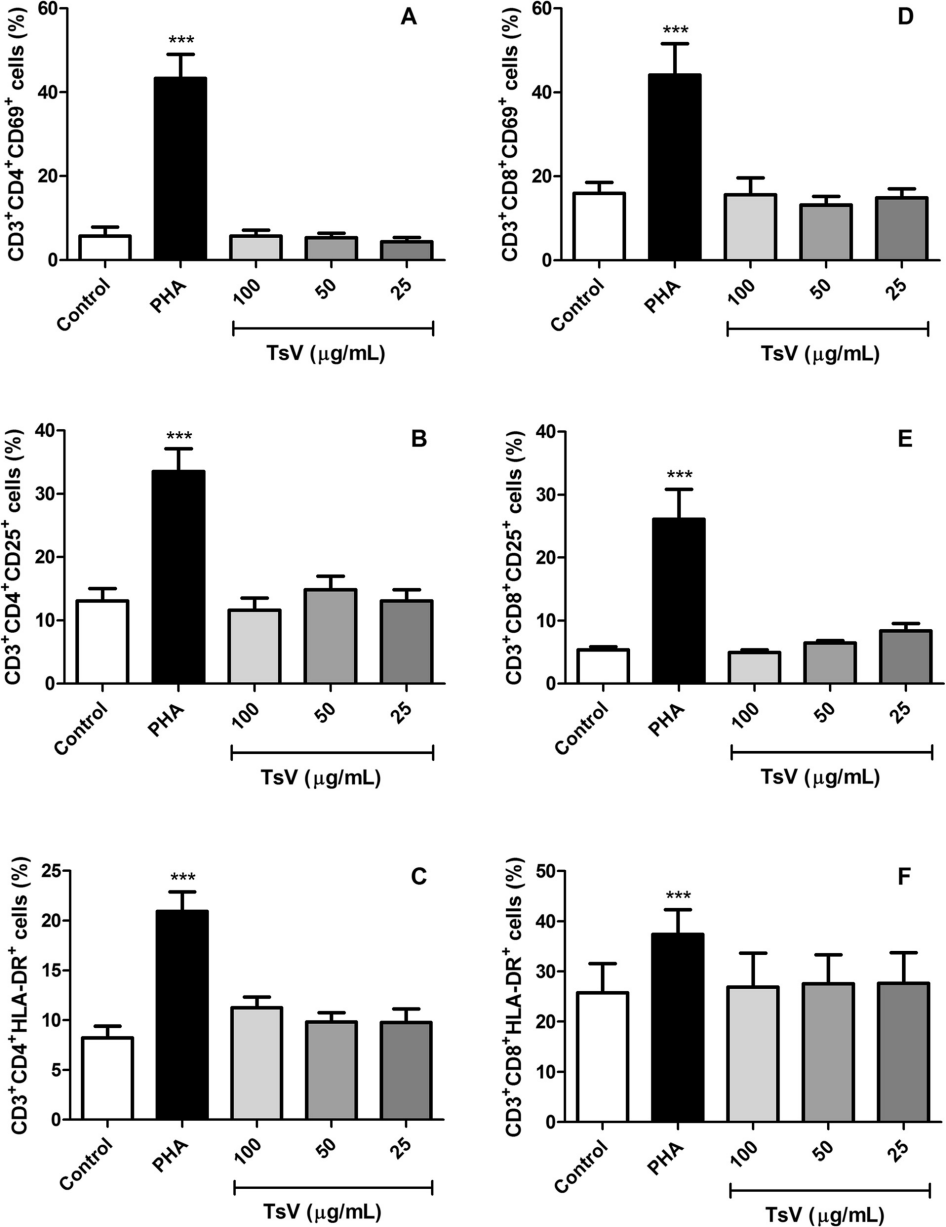
Lymphocyte subsets and their expression levels of activation markers after TsV treatment. Peripheral blood mononuclear cells (2 × 10^5^ cells) were cultured with *Tityus serrulatus* scorpion venom (TsV; 25, 50, and 100 μg/mL) or phytohemagglutinin (PHA; 2 μg/mL; positive control) for 24 h, at 37 °C, under 5 % CO_2_, and further stained with monoclonal antibodies to simultaneously detect CD3^+^CD4^+^ (**a**, **b**, **c**) and CD3^+^CD8^+^ (**d**, **e**, **f**) T lymphocyte subsets, and the expression of three activation markers: CD69 (**a**, **d**), CD25 (**b**, **e**), and HLA-DR (**c**, **f**). Control: untreated cells. Data are expressed as mean ± SEM of six experiments. ***p <0.001 vs. control

After 24 h of culture, TsV at concentrations of 25, 50, and 100 μg/mL did not induce significant changes in TCD3^+^CD4^+^ and CD3^+^CD8^+^ lymphocyte subpopulations, and did not modulate the expression of three activation markers – CD69, CD25 and HLA-DR – in PBMC (Fig. 2).

### TsV inhibits the proliferation of CD8^+^CD25^+^ T lymphocytes

**Fig. 3:**
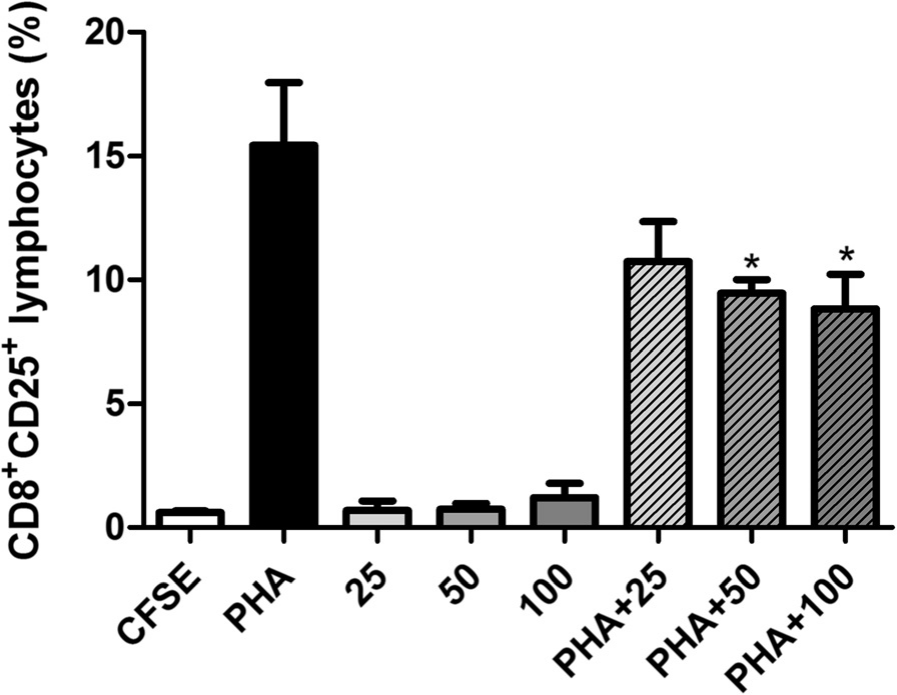
Percentage of CD8^+^ T lymphocytes expressing CD25. Peripheral blood mononuclear cells (PBMC; 5 × 10^6^cells/mL) were labeled with carboxyfluorescein diacetate succinimidyl ester (CFSE; 2.5 μM) and cultured with *Tityus serrulatus* scorpion venom (TsV; 25, 50, and 100 μg/mL) alone or in combination with phytohemagglutinin (PHA; 2 μg/mL) for 96 h at 37 °C, and under 5 % CO_2._ Then, PBMC were collected, labeled with anti-CD3/PerCP, anti-CD8/PE, and anti-CD25/APC monoclonal antibodies, and further analyzed by flow cytometry. CFSE: CFSE-stained cells cultured in RPMI; PHA: PHA-stimulated cells (positive control); 25, 50, 100: cells incubated with 25, 50, and 100 μg/mL TsV, respectively; PHA + 25, PHA + 50, PHA + 100: cells incubated with PHA plus 25, 50 and 100 μg/mL TsV, respectively. Each column represents the mean ± SEM of five experiments.*p < 0.05 vs. PHA

In PBMC previously stimulated with PHA, the 96-h treatment with TsV at 25, 50 or 100 μg/mL reduced the percentage of CD8^+^CD25^+^ lymphocytes by 35.7, 45.5, and 53.5 %, respectively, i.e. in a concentration-dependent manner (Fig. 3). These values were statistically different from the respective control (PHA) at TsV concentrations of 50 and 100 μg/mL (Fig. 3). In contrast, TsV did not alter either T lymphocyte proliferation extent or the percentage of CD3^+^CD4^+^ and CD3^+^CD4^+^HLA-DR^+^ lymphocyte subsets in non-stimulated PBMC, as compared with PHA- and PHA + TsV-stimulated PBMC (data not shown).

### TsV induces IL-6 release by PBMC

**Fig. 4:**
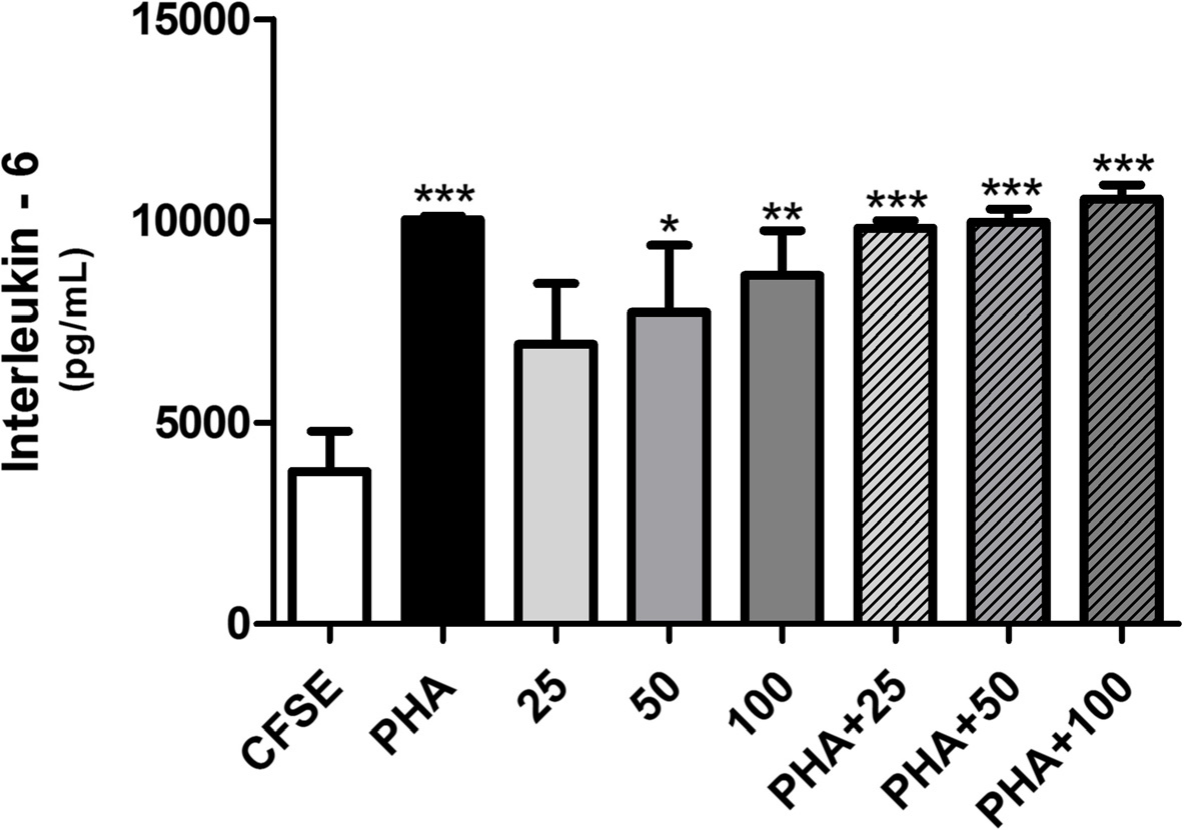
Interleukin-6 (IL-6) release by PBMC treated with *Tityus serrulatus* scorpion venom (TsV) for 96 h. Peripheral blood mononuclear cells (PBMC; 5.0 × 10^5^ cells) were labeled with carboxyfluorescein diacetate succinimidyl ester (CFSE; 2.5 μM) and cultured with TsV (25, 50, and 100 μg/mL) alone or in combination with phytohemagglutinin (PHA; 2 μg/mL) for 96 h. The levels of IL-6 in culture supernatants were determined by flow cytometry. CFSE: CFSE-stained cells cultured in RPMI 1640; PHA: PHA-stimulated cells (positive control); 25, 50, 100: cells incubated with 25, 50, and 100 μg/mL TsV, respectively; PHA + 25, PHA + 50, PHA + 100: cells incubated with PHA plus 25, 50, and 100 μg/mL TsV, respectively. Each column represents the mean ± SEM of five experiments. *p < 0.05, **p < 0.01, ***p < 0.001 vs. CFSE

TsV induced IL-6 release by PBMC in a concentration-dependent manner. The IL-6 levels in culture supernatants of non-stimulated PBMC were significantly increased at TsV concentrations of 50 and 100 μg/mL (*p* < 0.05 and *p* < 0.01, respectively), compared with the respective control (CFSE) (Fig. 4). TsV did not alter IL-6 release in PHA-stimulated cells, or the release of other cytokines (IL-2, IL-4, IL-10, IL-17, IFN-γ, and TNF-α) by non-stimulated and PHA-stimulated PBMC (data not shown).

## Discussion

In Brazil, the yellow scorpion *T. serrulatus* is the most dangerous Buthidae species and the main cause of serious accidents [4]. Its venom (TsV) contains toxins that act on K^+^ and Na^+^ channels, a property that accounts for great portion of the venom’s toxic effects. Several studies have reported that scorpion venoms and toxins, including TsV, elicit macrophage activation [11–13, 15, 19]. TsV and Ts1 bind Toll-like receptors to mediate cytokine and lipid mediator production [14]. Such reports have helped to elucidate how inflammatory mediators are produced after envenomation.

To date, very little is known about the direct effect of scorpion venoms on human lymphocyte functions. As lymphocytes have membrane K^+^ channels and TsV contains toxins that interact with these channels, we hypothesized that TsV and its toxins could affect lymphocyte functions mediated by the activity of K^+^ channels, such as PHA-induced cell proliferation [11, 20–22].

Cell stimulation by pathogens in infectious and inflammatory processes and by mitogens induces the expression of activation markers in T, B, and NK cells. PHA, a commonly used mitogen, is a plant lectin that binds to TCR and activates T lymphocytes [23]. In the present study, we examined the expression of very early (CD69), middle-to-late (CD25), and late (HLA-DR) T cell surface activation markers. CD69 expression can be detected within 1 to 2 h after T cell stimulation [24]. It can initiate tyrosine kinase and calcium flux activity, and transcription of IL-2 and TNF-α [25]. The expression of CD25 (the α-chain of the IL-2 receptor) and HLA-DR (a human class II major histocompatibility complex antigen) increases progressively after 24 h of stimulation with PHA [26].

After establishing the TsV concentrations and incubation time with PBMC, we examined whether TsV alters the percentage of T lymphocytes, and the extent of CD4^+^ and CD8^+^ T cell proliferation. In the presence of TsV, the percentage of T lymphocytes (data not shown), as well as the percentage of CD3^+^CD4^+^ and CD3^+^CD8^+^ lymphocyte subpopulations expressing CD69, CD25, and HLA-DR were similar to the untreated controls. However, maximum expression of some activation markers, especially CD25 and HLA-DR, may occur later. It suggests that the PBMC culture periods used (24 h and 96 h) were not sufficient to detect significant alterations in the expression level of these markers. To confirm this hypothesis, it is necessary to perform additional experiments using longer culture times. Another possibility is to use higher TsV concentrations to better evaluate its action on the expression of lymphocyte activation markers.

To assess lymphocyte proliferation, we selected CFSE due to its compatibility with several other fluorochromes, which enabled us to simultaneously evaluate several parameters including the percentage of activated cells and proliferation of distinct populations of activated cells, using multi-color flow cytometry [27]. With the aid of this technique, we found that TsV at 50 and 100 μg/mL significantly decreased the percentage of CD8^+^CD25^+^ T cells in PHA-stimulated PBMC (Fig. 3). Considering that ion channels participate in an early stage of cell activation by PHA, it is possible that toxins that exist in TsV act on ions channels in CD8^+^CD25^+^ T cells and thereby impair the action of PHA on these cells [22]. It is well documented that ion channels play important roles in the activation and proliferation of lymphocytes, as well as in the production of cytokines [20, 28, 29]. Blockage of the K^+^ channel Kv1.3 inhibits mitogen-induced T cell proliferation, protein synthesis and IL-2 production [20].

Cytokines are produced during the effector phases of innate and acquired immune responses and regulate immune and inflammatory responses [30, 31]. Besides the knowledge that TsV and its toxins are potent inducers of cytokine and lipid mediator production in vitro and in vivo [11–15, 19], little is known about the effect of TsV on the production of cytokines by human PBMC. We measured the levels of various cytokines – IL-2, IL-4, IL-6, IL-10, IL-17, IFN-γ and TNF-α – in the supernatant of PBMC cultures after 96 h of treatment with PHA and/or different concentrations of crude TsV. Compared with untreated cells, TsV at 50 and 100 μg/mL significantly increased IL-6 release in non-stimulated cells (*p* < 0.05 and *p* < 0.01, respectively) (Fig. 4). In contrast, TsV did not alter the PHA-induced IL-6 production by PBMC. Our results partially agree with those reported by several in vitro and in vivo studies [11–15, 19]. TsV fractions stimulate the macrophage production of proinflammatory cytokines such as TNF-α, IL-1, and IL-6 [32]. TsV and the toxins Ts1 and Ts6 stimulate the production of NO, IL-6, and TNF-α in J774.1 cells [12]. When inoculated in mice, Ts2 and Ts6 induce the production of the proinflammatory cytokines IL-6, TNF-α, IL-1β, and IFN-γ, and the lipid mediators prostaglandin E2 and leukotriene B4 [13]. Several authors have reported that IL-6 levels are increased after envenomation in humans [9, 33–35].

To the best of our knowledge, this is the first study that shows immunomodulatory actions of TsV on human T lymphocytes. Taken together, our results suggest that TsV is a potential source of molecules with immunomodulatory effects on these cells, and should stimulate further research to elucidate the action mechanisms involved, including the participation of T lymphocyte ion channels in the phenomena observed.

## Conclusions

TsV is a potential immunomodulator of human T lymphocyte functions. The results reported herein stimulate further investigations to identify the venom components responsible for the observed effects and to elucidate the molecular mechanisms involved in TsV immunomodulatory action on lymphocytes. These investigations may facilitate the development of new tools to study the pathophysiological mechanisms of envenomation and to discover new treatment alternatives for diseases mediated by the immune system.

### Ethics committee approval

The present study was approved by the Human Research Ethics Committee at FCFRP-USP registered under number CEP/FCFRP 166.

## Additional file

Additional file 1:
**Representative picture showing forward/side scatter** **dot-plot** **of human PBMC treated with TsV.** Peripheral blood mononuclear cells (PBMC; 2 × 10^6^ cells/mL) were cultured with *Tityus serrulatus* scorpion venom (TsV; 25, 50, and 100 μg/mL), phytohemagglutinin (PHA; 2 μg/mL; positive control) for 24 h, at 37 °C, and under 5 % CO_2_. Untreated PBMC represents the negative control. PBMC were labeled with anti-CD3/FITC and anti-CD8/PE monoclonal antibodies and further analyzed by flow cytometry. The lymphocyte gate was selected and analyzed to calculate the percentage of stained cells. The figure depicts a representative analysis from six independent experiments. The percentages in parentheses refer to CD3+ CD8+ cells (P2 region). (A) Negative control (5.5 %). (B) PHA-stimulated cells (26.1 %). (C) 100 μg/mL of TsV (4.9 %). (D) 50 μg/mL of TsV (6.4 %). (E) 25 μg/mL of TsV (8.4 %). (DOCX 204 kb)
