# Supplementary material for: Immunomodulatory activity of Tityus serrulatus scorpion venom on human T lymphocytes
Source: J Venom Anim Toxins Incl Trop Dis. 2015 Nov 11;21:46. doi: 10.1186/s40409-015-0046-3 (PMC4642687; doi:10.1186/s40409-015-0046-3)
Supplement: Additional file 1: — Representative picture showing forward/side scatter dot-plot of human PBMC treated with TsV. Peripheral blood mononuclear cells (PBMC; 2 × 10^6^ cells/mL) were cultured with Tityus serrulatus scorpion venom (TsV; 25, 50, and 100 μg/mL), phytohemagglutinin (PHA; 2 μg/mL; positive control) for 24 h, at 37 °C, and under 5 % CO2. Untreated PBMC represents the negative control. PBMC were labeled with anti-CD3/FITC and anti-CD8/PE monoclonal antibodies and further analyzed by flow cytometry. The lymphocyte gate was selected and analyzed to calculate the percentage of stained cells. The figure depicts a representative analysis from six independent experiments. The percentages in parentheses refer to CD3+ CD8+ cells (P2 region). (A) Negative control (5.5 %). (B) PHA-stimulated cells (26.1 %). (C) 100 μg/mL of TsV (4.9 %). (D) 50 μg/mL of TsV (6.4 %). (E) 25 μg/mL of TsV (8.4 %). (DOCX 204 kb) [file 40409_2015_46_MOESM1_ESM.docx]

**Additional file 1**

**Supplementary Figure 1**
